# Supplementary material for: Control of inorganic and organic phosphorus molecules on microbial activity, and the stoichiometry of nutrient cycling in soils in an arid, agricultural ecosystem
Source: PeerJ. 2024 Sep 23;12:e18140. doi: 10.7717/peerj.18140 (PMC11426319; doi:10.7717/peerj.18140)
Supplement: Supplemental Information 2 — a Abbreviations: AMP, adenosine monophosphate; and MAP, monoammonium phosphate. BG, β-glucosidase; NAG, N-acetyl glucosaminidase; POX, Polyphenol oxidase (laccase; Phm, Phosphomonoesterase; Phd, Phosphodiesterase; Phy, Phytase. Note: Different uppercase letters (A, B, C, D) within the same row indicate significant differences among different treatments as obtained from the Tukey test. Standard error is shown in parentheses. bSignificance values are represented with an “*”, where *, p < 0.05; ** p < 0.01; ***, p < 0.001. [file peerj-12-18140-s002.docx]

Table S1. Means and standard error of enzyme activities measured for each treatment after the 19 days of the incubation experiment.

| **Variable** | **Control** | **MAP**  **(Pi)** | **Ca(H_2_PO_4_)_2_**  **(Pi)** | **RNA**  **(Po)** | **AMP**  **(Po)** | **Phytic acid**  **(Po)** | **p** |
| --- | --- | --- | --- | --- | --- | --- | --- |
| BG (µmol pnp h^-1^ g ^-1^) | 0.086 (±0.02) | 0.059 (±0.012) | 0.053 (±0.015) | 0.056 (±0.015) | 0.08 (±0.028) | 0.051 (±0.018) | 0.673 |
| POX (µmol tyr h^-1^ g ^-1^) | 0.3(±0.031) | 0.181 (±0.061) | 0.093 (±0.06) | 0.306 (±0.028) | 0.206 (±0.098) | 0.302 (±0.072) | 0.129 |
| NAG (µmol pnp h^-1^ g ^-1^) | 0.02 (±0.008) ^AB^ | 0.027 (±0.004) ^AB^ | 0.032 (±0.008) ^A^ | 0.003 (±0.001) ^B^ | 0.001 (±0.001) ^B^ | 0.023 (±0.01) ^AB^ | 0.0079** |
| Phm (µmol pnp h^-1^ g ^-1^) | 0.016(±0.008) | 0.023 (±0.013) | 0.186 (±0.157) | 0.041 (±0.006) | 0.052 (±0.028) | 0.04 (±0.017) | 0.486 |
| Phd (µmol pnp h^-1^ g ^-1^) | 0.212(±0.021) | 0.172 (±0.013) | 0.174 (±0.014) | 0.262 (±0.091) | 0.258 (±0.029) | 0.224 (±0.009) | 0.477 |
| Phy (µmolpi h^-1^ g ^-1^) | 1.085(±0.32) | 0.464 (±0.251) | 0.87 (±0.476) | 0.418 (±0.182) | 0.665 (±0.311) | 0.361 (±0.225) | 0.521 |

^a^Abbreviations: AMP, adenosine monophosphate; and MAP, monoammonium phosphate. BG, β-glucosidase; NAG, N-acetyl glucosaminidase; POX, Polyphenol oxidase (laccase; Phm, Phosphomonoesterase; Phd, Phosphodiesterase; Phy, Phytase. Note: Different uppercase letters (A, B, C, D) within the same row indicate significant differences among different treatments as obtained from the Tukey test. Standard error is shown in parentheses.

^b^Significance values are represented with an “*”, where *, p<0.05; ** p<0.01; ***, p<0.001.
