# Supplementary material for: Control of inorganic and organic phosphorus molecules on microbial activity, and the stoichiometry of nutrient cycling in soils in an arid, agricultural ecosystem
Source: PeerJ. 2024 Sep 23;12:e18140. doi: 10.7717/peerj.18140 (PMC11426319; doi:10.7717/peerj.18140)
Supplement: Supplemental Information 3 [file peerj-12-18140-s003.docx]

Table S2. Percent of variance explained by the first two components (Eigenvalue) and weight of analized variables in the two first components (Eigenvector).

|  | **PC 1** | **PC 2** |
| --- | --- | --- |
| *Eigenvalue* | 3.69 | 2.32 |
| Percent (%) | 26.36 | 16.57 |
| Accumulated percent (%) | 26.36 | 42.93 |
|  | ***Eigenvector of the variables*** | |
| DOC | -0.74 | -0.0074 |
| DON | 0.082 | 0.46 |
| DOP | -0.69 | 0.476 |
| Cmic | -0.557 | -0.263 |
| Nmic  Pmic | 0.482  0.74 | -0.436  0.026 |
| NO_3_ | 0.802 | -0.22 |
| PO_4_ | -0.048 | 0.676 |
| Phm | -0.049 | 0.125 |
| Phd | 0.439 | -0.021 |
| Phy | -0.468 | -0.473 |
| NAG  BG  POX | -0.707  -0.074  0.045 | 0.457  -0.416  -0.704 |
